# A non-randomized, open-label study to assess the impact of rounds of mass drug administration with artemisinin-piperaquine plus primaquine on malaria in São Tomé Island

**DOI:** 10.1186/s13071-025-06768-1

**Published:** 2025-05-16

**Authors:** Mingqiang Li, Ruixiang Tan, Peiting Chen, Herodes Sacramento Rampao, Carlos Alberto Bandeira D’almeida, Guoming Li, Jingwen Liu, Guozhuang Shi, Fei Chen, Lei Shu, Xinan Huang, Changsheng Deng, Wenfeng Guo, Jianping Song

**Affiliations:** 1 Artemisinin Research Center, Guangzhou University of Chinese Medicine (GZUCM), Guangzhou, 510405 People’s Republic of China; 2 Centro Nacional de Endemias, Ministry of Health, São Tomé, 100600 São Tomé and Príncipe; 3 School of Public Health and Management, Guangzhou University of Chinese Medicine (GZUCM), Guangzhou, 510405 People’s Republic of China; 4 Science and Technology Park, Guangzhou University of Chinese Medicine (GZUCM), Guangzhou, 510405 People’s Republic of China

**Keywords:** Malaria, Mass drug administration (MDA), Artemisinin-piperaquine (AP), Primaquine (PMQ), *P. falciparum*

## Abstract

**Background:**

The aim of this study was to explore the effect of mass drug administration (MDA) on malaria transmission in low-endemic malaria areas.

**Methods:**

Mass drug administration of artemisinin-piperaquine (AP) + primaquine (PMQ) was targeted to 17,438 individuals in the Agua Grande region of São Tomé and Príncipe (STP). The participants were allocated to either a three-round MDA (3-MDA) group or to a two-round MDA (2-MDA) group. The coverage rate, compliance, adverse events and other indicators were evaluated.

**Results:**

Mass drug administration coverage rate in the 3-MDA group (20,548 person-times) was 84.23–89.14%, with a compliance of 68.38%. MDA coverage rate in the 2-MDA group (15,365 person-times) was 87.30–93.23%, with a compliance of 80.70%. The rates of MDA-related adverse reactions were low in both the 3-MDA (0.75%) and 2-MDA (0.72%) groups, and no serious adverse reactions were observed. Malaria incidence decreased by 80.47% (*z* = − 2.35, *P* = 0.019) and 72.27% (*z* = − 0.89, *P* = 0.372) in the 3-MDA and 2-MDA groups, respectively, within 1 year.

**Conclusions:**

Two or three rounds of MDA with AP and PMQ in STP safely and rapidly reduced the prevalence of malaria cases and infections. It is possible that two rounds of MDA in certain districts may achieve the desired outcomes.

**Graphical Abstract:**

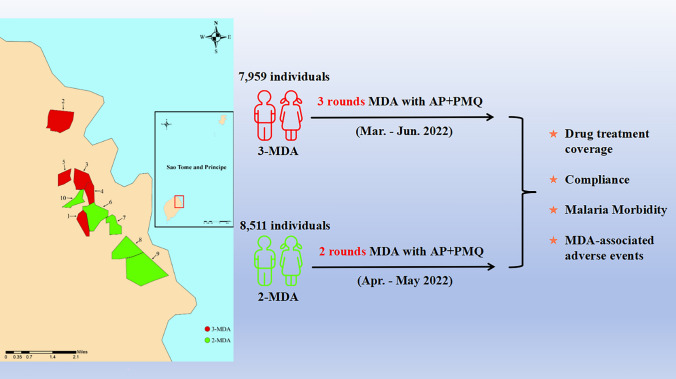

**Supplementary Information:**

The online version contains supplementary material available at 10.1186/s13071-025-06768-1.

## Background

The coronavirus disease 2019 (COVID-19) pandemic in 2020 has resulted in the resurgence of the malaria epidemic in Africa. São Tomé and Príncipe (STP), a small African country, has a significant malaria burden, reflecting the overall trend across the African continent [1]. According to the National Centre for Disease Control and Prevention (NCDC) of STP, the total number of malaria cases in the country in 2022 was 3979, representing an increase of 104.68% and 45.75% compared to 2020 (1944 cases) and 2021 (2730 cases), respectively [2]. The significant rise in malaria cases has been predominantly observed in the Agua Grande region, where the capital, São Tomé, is situated. This area serves as the political and cultural hub of the country, housing a substantial portion of the population, Therefore, not surprisingly, this area accounts for more than one-third of the country's malaria cases and also represents the largest residual focus in STP. This development has prompted the Government of STP to increase its efforts to combat malaria.

Mass drug administration (MDA) represents a potential strategy to eliminate *Plasmodium falciparum* infections and reduce the human parasite reservoir. MDA often requires extensive collaboration from all community members, irrespective of their infection status, together with the administration of a complete regimen of anti-malarial drugs [3–5]. Asymptomatic infections serve as significant reservoirs for malaria parasites, contributing substantially to the persistence of malaria transmission within populations [6, 7]. It is known that in malaria-endemic areas, especially those with low transmission prevalence, a persistent population of asymptomatically infected individuals exists. These individuals do not show clinical symptoms of malaria but frequently carry *P. falciparum* gametocytes [8, 9]. Several studies have shown that primaquine (PMQ) is effective in killing *P. falciparum* gametocytes [10–12], with a single dose of PMQ (0.25 mg/kg), administered without testing for glucose-6-phosphate dehydrogenase (G6PD) deficiency, effectively clearing *P. falciparum* gametocytes while minimizing the severe complications associated with G6PD deficiency [13].

For this reason, the addition of a single dose of PMQ to MDA programs could eliminate* P. falciparum* gametocytes in humans, disrupting human-mosquito transmission and preventing further spread of malaria [14]. Various studies [15–18] have shown that MDA can have an immediate effect in areas of high transmission intensity, such as Comoros [18, 19], Cambodia [20], Papua New Guinea [17] and the Gambia [16]. Eisele et al. [21] reported that two rounds of MDA rapidly reduced infection prevalence, infection incidence and confirmed case incidence rates, especially in low transmission areas.

To rapidly eliminate *P. falciparum* from residual active foci, interrupt national malaria transmission and reduce the disease incidence, an strategy was adopted together with the Ministry of Health of STP to carry out MDA with artemisinin-piperaquine (AP) + PMQ from March to June 2022 in 10 villages with high malaria prevalence in the Agua Grande Region. This study was designed to undertake two or three rounds of MDA to assess the impact of the number of rounds on coverage and adherence as well as compare the adverse effects, rates of parasitemia and *P. falciparum* gametocytemia among children and malaria case incidence of the two regimens.

## Methods

### Study sites

**Fig. 1:**
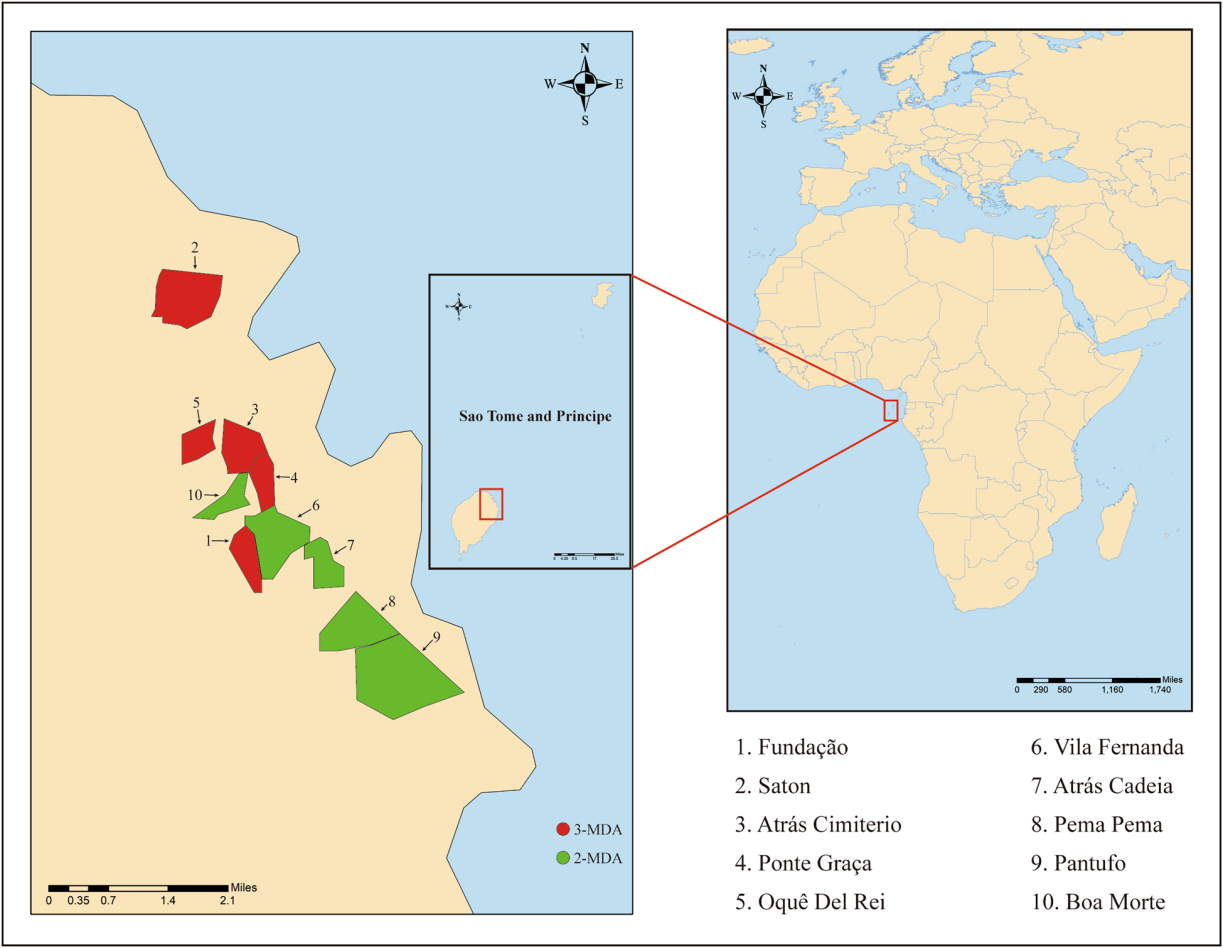
Location of study sites in São Tomé Island. 3-MDA, Three rounds of MDA; 2-MDA, Two rounds of MDA; MDA, mass drug administration

The 10 villages of STP included in this study were from the Agua Grande Region and, from north to south, were Saton, Oquê Del Rei, Atrás Cimiterio, Ponte Graça, Boa Morte, Vila Fernanda, Fundação, Atrás Cadeia, Pema Pema and Pantufo. The geographical location of these villages is shown in Fig. 1. These villages were selected for inclusion in the study based on their geographical location, population and malaria incidence during the last 3 years. Three rounds of MDA (3-MDA) were conducted in five villages (i.e. Fundação, Saton, Atrás Cimiterio, Ponte Graça and Oquê Del Rei) in the north of the region, and two rounds of MDA (2-MDA) were conducted in the remaining five villages in the south of the region (i.e. Vila Fernanda, Atrás Cadeia, Pema Pema, Pantufo and Boa Morte) (Additional file 1: Table S1).

### Study design

#### Protocol

**Fig. 2:**
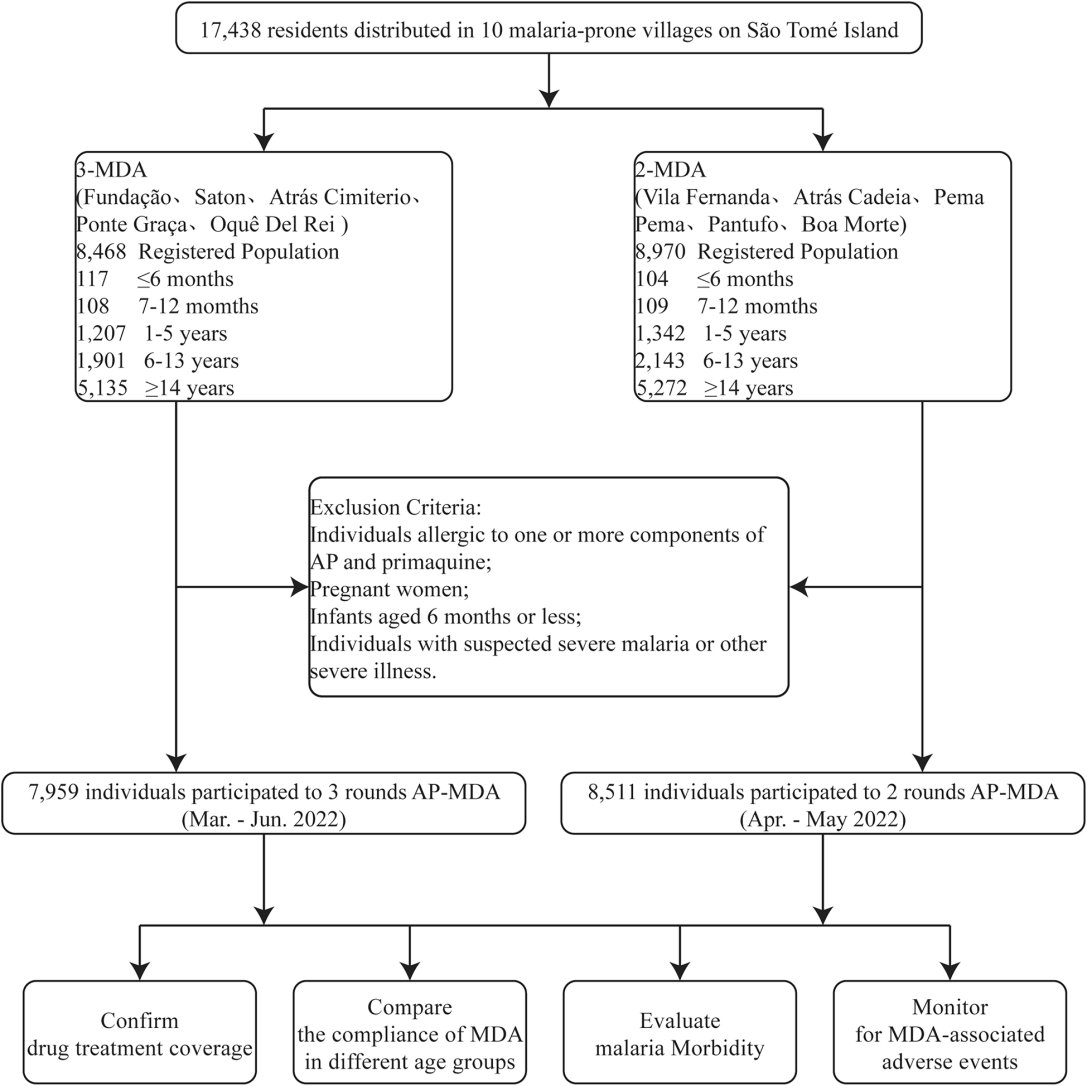
Study execution scheme. AP, Artemisinin-piperaquine; 2-MDA, two rounds of MDA; 3-MDA, three rounds of MDA; MDA, mass drug administration

The first phase of the trial has been published earlier [15]. In brief, according to the protocol approved, the participants were allocated to either a 3-MDA group or a 2-MDA group, and there was, in principle, a 28-day interval between each round of MDA. The first round of the 3-MDA arm of the study occurred between 28 March 28 and 8 May 2022; the second round was from 9 May to 5 June 2022; and the third round was from 6 June to 4 July 2022. The first round of the 2-MDA arm of the study was held from 19 April to 22 May 2022, and the second round was from 23 May to 20 June 2022. The second phase of this trial was approved by the ethics committees of the Centro Nacional de Endemias of São Tomé and Principe (No. 38/GMS/2022) and the Guangdong Provincial Hospital of Chinese Medicine (No. BF2019-055–01). Informed consent was obtained from all participants enrolled in the study (Fig. 2). Similar to the study design in the first phase, a top-down model was adopted for the division of labor and management [22]. It also added training in the use of PMQ and the management of adverse reactions after taking PMQ.

#### Inclusion and exclusion criteria

Inclusion criteria were (i) permanent residency at a study site; (ii) age ≥ 6 months; and (iii) signed consent form (provided by participant or by participant’s guardian) to participate in the study. Individuals were excluded from the study if they: (i) had a previous history of allergy to drugs, especially to ≥ 1 components of AP and PMQ; (ii) were pregnant; (iii) aged < 6 months; and (iv) were suspected of having severe malaria or another severe illness.

### Dosage and frequency of medication

The AP tablets used in the present study (Artequick; Artepharm Co., Ltd., China) were the same as those used in the first phase of the study. The tablets were taken once a day for 2 consecutive days [15], with one dose administered on day 1 and one dose administered on day 2 at the start of each round of MDA. A single dose of PMQ (7.5 mg/tablet; Ipca Laboratories Ltd., Maharashtra, India), administered by a technician at a dose of 0.25 mg/kg, was used on day 1 of each round of MDA. Participants in the 3-MDA arm of the study did not take PMQ in round 3 due to delays in PMQ shipments, which resulted in the drug not arriving promptly. All tablets were given orally to each participant. If a patient took less than one full tablet, the technician used a pill cutter to cut the tablet before giving it to the patient (Additional file 2: Table S2).

### Estimates of parasitemia and gametocytemia

All participants were tested using rapid diagnostic tests (RDTs) and blood smears before the start of MDA. At 6 months post-MDA, 100 children aged 6 months to 10 years from different locations in each village were selected for testing using RDTs and blood smears. The prevalence of parasitemia and gametocytemia was calculated based on the results of the microscopy tests in the village in general.

### Adverse reaction monitor

All participants were instructed to avoid taking AP and PMQ tablets on an empty stomach. During the MDA, one to two health workers from the Regional Hospital of Agua Grande were assigned to each village to handle adverse drug reaction events and monitor participants for 4 days. All adverse reactions were recorded on an Adverse Reaction Form (Additional file 3: Table S3).

### Laboratory tests

All thick and thin blood smears were collected from a fingertip blood sample. These smears were sent to the laboratory of the Regional Hospital of Agua Grande for analysis and were subsequently stained with a 10% Giemsa solution and examined by a certified malaria microscopist. Smears were considered negative if no parasites were detected in 200 fields using an oil-immersion lens (magnification 100×). About 10% of the smears were randomly selected and rechecked by another microscopist to determine the quality of the results. If the results were inconsistent, a consensus was reached through discussion involving a third microscopist.

### Follow-up

All participants with malaria were required to complete a 28-day follow-up according to the local malaria guidelines in STP. On days 0, 1, 2, 3, 7, 14, 21 and 28 after treatment, the patients were observed for clinical signs and blood smears were studied to identify the* Plasmodium* species and determine density counts.

### Data collection

The baseline data for the 10 villages were collected by field technicians, including name, gender, age, pregnancy, history of allergies, history of diseases and house number. Each participant was tested for malaria using RDTs, and two blood smears were made. All results were counted, collated and stored by the NCDC statistician. The technicians also collected information on the geographical coordinates of each village for future mapping purposes. During MDA, the field technicians documented the medication administration for all villagers using a medication register, while doctors recorded the adverse events. The number of malaria cases by month and region is available from the NCDC.

### Statistical analysis

In this study, the seasonal Mann–Kendall test was used to examine trends in the incidence of malaria. The Joinpoint Regression Program (version 4.9.1.0) was employed to determine both the number and precise locations of inflection points utilizing the Grid Search Model (GSM) alongside the Monte Carlo permutation test. Variations in trends within segments and across the entire dataset were reflected through the computation of monthly percent change (MPC), average monthly percent change (AMPC) and their corresponding 95% confidence intervals for both designated regions. A *P*-value of < 0.05 was considered to be statistically significant.

The Statistical Package for the Social Sciences V. 26 (SPSS IBM Corp., Armonk, NY, USA) was used for the statistical analysis of the data. Qualitative indicators were presented using percentages or composition ratios for descriptive purposes, and quantitative indicators were presented using means and standard deviations. The chi-square (*χ*^2^) test or Fisher's exact test was used for comparing two groups. The statistical significance level was defined as a two-sided *P*-value of < 0.05.

## Results

### Baseline information

The total registered population for the 3-MDA and 2-MDA groups was 17,438, with males accounting for 46.34% and 45.08% of each group, respectively. Age stratification results indicated no statistically significant differences between groups in the number of infants (age groups < 6 months and 6–12 months) and children (age group 1–5 years) (*P* > 0.05). In contrast, the number of individuals in the age groups 6–13 years and ≥ 14 years was higher in the 2-MDA group than in the 3-MDA group (*P* = 0.025; *P* = 0.012). There was no statistically significant difference in the number of pregnant women and individuals with severe diseases (*P* > 0.05). Pre-MDA malaria screening results showed that 102 cases were positive by RDT, with a higher number of positives in the 3-MDA (*n* = 61) group than in the 2-MDA (*n* = 41 group) (*P* = 0.028). The laboratory microscopy studies detected 37 malaria-positive cases (23 *P. falciparum* gametocytes), with no statistically significant difference between the MDA groups (*P* > 0.05) (Additional file 4: Table S4).

### MDA coverage rate

A total of 35,913 person-times participated in the MDA, with 3-MDA having 6704, 6749 and 7095 person-times across its three rounds, totaling 20,548 person-times. The 2-MDA had 7430 and 7935 person-times across its two rounds, totaling 15,365 person-times (Table 1). However, during all rounds of the 3-MDA and 2-MDA, 7431 individuals did not participate. In the 3-MDA group, 1764, 1719 and 1373 individuals did not participate in the first, second and third rounds, respectively, while in the 2-MDA group, 1540 and 1035 individuals did not participate in the first and second rounds, respectively. The main reasons cited for non-participation were: infants (aged < 6 months; 221 individuals), pregnancy (442 women), severe diseases (60 individuals), travel or overseas work (245 individuals) and refusal to participate in the study (27 individuals) (Additional file 4: Table S4). The coverage rates for each round of the 3-MDA were 84.23%, 84.8% and 89.14%, respectively, while the coverage rate for participation in two or more rounds of the 3-MDA was 91.59%, and the coverage rate for participation in all three rounds was 68.38%. The coverage rates for each round of the 2-MDA were 87.30% and 93.23%, respectively, and the coverage rate for participation in both rounds of the 2-MDA was 80.70%.

**Table 1:** Mass drug administration Coverage Rate (rounds)

MDA rounds and District	Number of individuals in the registered population	Number of individuals taking medicine, % (*n*/*N*)				
		Round 1	Round 2	Round 3	Two or more rounds	Three rounds
*3-MDA*						
Fundação	944	82.04 (731/891)	89.90 (801/891)	92.37 (823/891)	94.73 (844/891)	69.81 (622/891)
Saton	1050	73.29 (741/1011)	88.63 (896/1011)	92.88 (939/1011)	92.68 (937/1011)	75.17 (760/1011)
Atrás Cimiterio	1127	80.97 (851/1051)	83.54 (878/1051)	91.72 (964/1051)	89.15 (937/1051)	67.75 (712/1051)
Ponte Graça	2068	87.33 (1717/1966)	83.32 (1638/1966)	88.96 (1749/1966)	94.61 (1860/1966)	65.06 (1279/1966)
Oquê Del Rei	3279	87.63 (2664/3040)	83.42 (2536/3040)	86.18 (2620/3040)	89.21 (2712/3040)	68.06 (2069/3040)
Total	8468	84.23 (6704/7959)	84.80 (6749/7959)	89.14 (7095/7959)	91.59 (7290/7959)	68.38 (5442/7959)
*2-MDA*						
Vila Fernanda	787	95.30 (710/745)	87.92 (655/745)	–	83.62 (623/745)	–
Atrás Cadeia	1290	84.28 (1040/1234)	94.98 (1172/1234)	–	79.42 (980/1234)	–
Pema Pema	1301	83.43 (1022/1225)	94.37 (1156/1225)	–	78.04 (956/1225)	–
Pantufo	2630	89.59 (2228/2487)	96.62 (2403/2487)	–	86.25 (2145/2487)	–
Boa Morte	2962	86.17 (2430/2820)	90.39 (2549/2820)	–	76.74 (2164/2820)	–
Total	8970	87.30 (7430/8511)	93.23 (7935/8511)	–	80.70 (6868/8511)	–

*2-MDA* Two rounds of MDA, *3-MDA* three rounds of MDA, *MDA* mass drug administration

### MDA-associated adverse events

In this study, a total of 265 adverse events (0.74%) related to MDA were reported, with 154 events (0.75%) occurring in the 3-MDA group and 111 events (0.72%) occurring in the 2-MDA group. The primary symptoms included headache (0.19%), dizziness (0.15%), fever (0.05%), nausea (0.02%), vomiting (0.09%) and abdominal pain (0.14%). Other symptoms (0.04%) included weakness, diarrhea, itching and muscle pain. All adverse events recovered spontaneously at the end of dosing, and no deaths or other serious adverse events were reported (Additional file 5: Table S5)

### Parasitemia and gametocytemia rates

All detected parasites were *P. falciparum*. The parasitemia rates for the 3-MDA group at the pre-MDA time point and at 6 months post-MDA were 2.24‰ (19/8468) and 0 (0/500), respectively. The gametocytemia rates were 1.30‰ (11/8468) and 0 (0/500) at these same time points, respectively.

The parasitemia rates for the 2-MDA group at the pre-MDA time point and at 6 months post-MDA were 2.01‰ (18/8970) and 2.00‰ (1/500), respectively. The gametocytemia rates were 1.34‰ (12/8970) and 0 (0/500) at these same time points, respectively (Additional file 6: Table S6).

### Malaria cases surveillance

According to malaria case data provided by the NCDC in STP for the period 2019–2023, the malaria incidence rates per 1000 population in the 3 years, 2 years and 1 year prior to the three rounds of MDA were 29.53 (239/8093), 21.3 (175/8216) and 41.12 (343/8341), respectively. One year after the three rounds of MDA, the malaria incidence rate per 1000 population decreased to 7.91 (67/8468), with reductions of 71.97%, 61.71% and 80.47% (*z* = − 2.35, *P* = 0.019) compared to 3 years, 2 years and 1 year before MDA (pre-intervention periods), respectively. Before the two rounds of MDA, the malaria incidence per 1000 population in the 3 years, 2 years and 1 year prior to intervention was 13.65 (117/8573), 11.26 (98/8703) and 28.97 (256/8836), respectively. One year after the two rounds of MDA, the incidence rate further decreased to 7.92 (71/8970), with reductions of 39.32%, 27.55% and 72.27% (*z* = − 0.89, *P* = 0.372) compared to 3 years, 2 years and 1 year before MDA (pre-intervention periods), respectively (Additional file 7: Table S7).

**Fig. 3:**
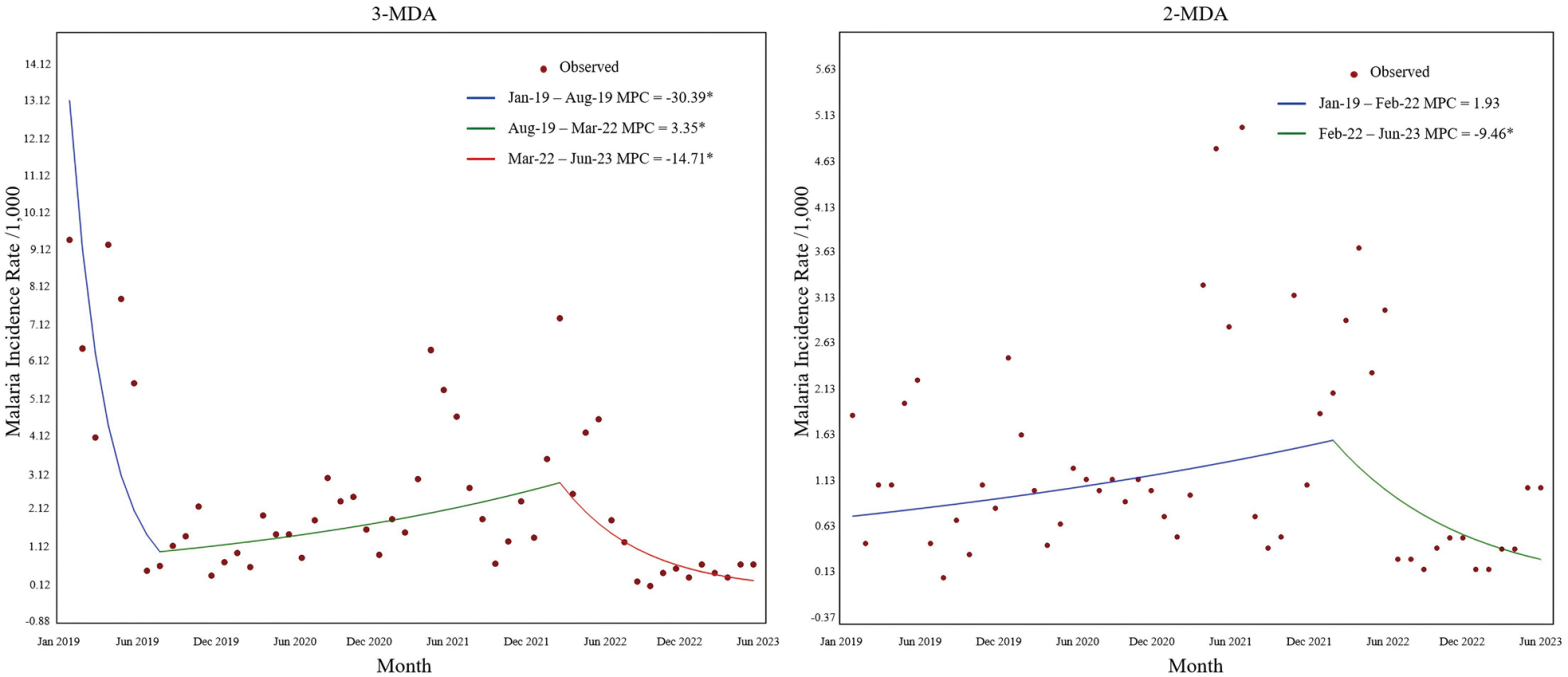
Joinpoint regression analysis of malaria incidence in two regions from January 2019 to June 2023. 2-MDA, Two rounds of MDA; 3-MDA, three rounds of MDA; MDA, mass drug administration; MPC, monthly percent change

A log-linear model for the analysis was used as the normality test results of malaria incidence rates in both the 2-MDA and 3-MDA regions were skewed (*P* < 0.001). The results of the Joinpoint regression for the 3-MDA region showed the existence of two turning points, namely August 2019 and March 2022 (Fig. 3a), with a significant downward trend in the overall incidence rate (AMPC: − 7.09, *t* = − 7.09, *P* < 0.01). The malaria incidence rates between January and August 2019 showed a significant decreasing trend (MPC = − 30.39, *t* = − 2.69, *P* < 0.05), a significant increase in incidence between August 2019 and March 2022 (MPC = 3.35, *t* = 2.19, *P* < 0.05) and a significant decrease between March 2022 and June 2023 (MPC = − 14.71, *t* = − 3.73, *P* < 0.01). The Joinpoint regression for the 2-MDA region showed a turning point only in February 2022 (Fig. 3b), with a downward trend in the overall data that was not statistically significant (AMPC = − 1.65, *t* = − 1.09, *P* = 0.277). There was an upward trend from January 2019 to February 2022, without a statistically significant difference (MPC = 1.93, *t* = 1.59, *P* = 0.117). However, a significant decrease in malaria incidence was observed from February 2022 to June 2023 (MPC = − 9.46, *t* = − 2.35, *P* < 0.05) (Additional file 8: Table S8).

**Fig. 4:**
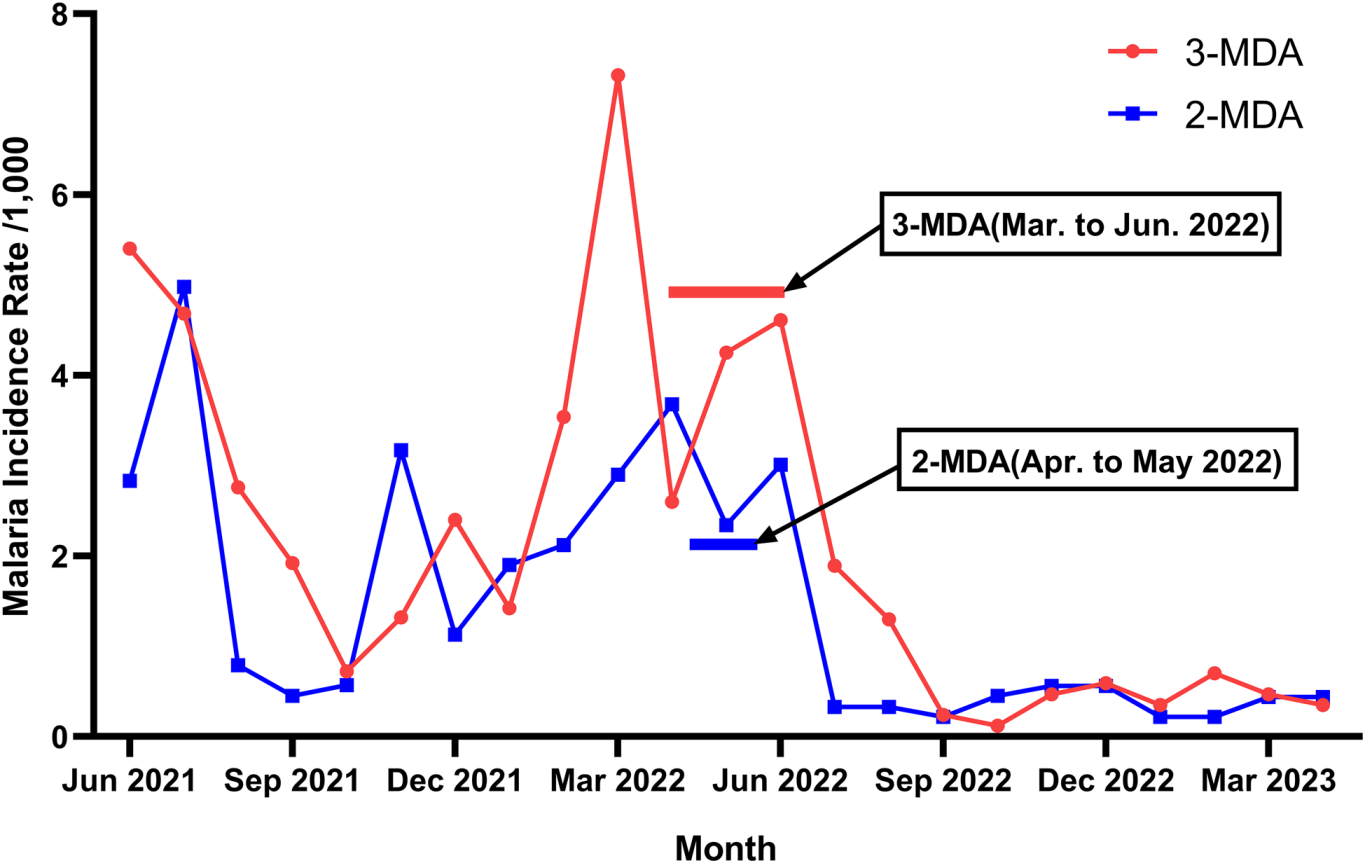
Incidence of malaria cases before and after MDA. 2-MDA, Two rounds of MDA; 3-MDA, three rounds of MDA; MDA, mass drug administration

The incidence of malaria cases was assessed every 3 months before and at various time points after the implementation of MDA (Fig. 4). It was observed that for the 3-MDA group, incidence of malaria per 1000 population at 3 months pre-MDA and at 3, 6, 9 and 12 months post-MDA was 12.28 (104/8468), 3.42 (29/8468), 1.18 (10/8468), 1.51 (13/8595) and 1.75 (15/8595), respectively, and that for the 2-MDA group, the incidence of malaria per 1000 population at these same time points was 6.91 (62/8970), 3.68 (33/8970), 1.23 (11/8970), 0.99 (9/9104) and 1.98 (18/9104), respectively (Additional file 9: Table S9). The results demonstrated that, compared to the 3 months pre-MDA, the 3-MDA intervention group showed reductions of 72.12%, 90.38%, 87.50%, and 85.58% in incidence rates, while the 2-MDA group exhibited decreases of 46.77%, 82.26%, 85.48%, and 70.97%, respectively.

## Discussion

The WHO recommends MDA as a potential strategy for eliminating *P. falciparum* malaria in areas approaching transmission interruption [4, 16]. Previous studies [15, 17, 18, 23–26] have shown that MDA once a month for three consecutive rounds of MDA can rapidly reduce malaria prevalence in the implementation area and provide a radiating protective effect to surrounding areas. STP (0°20′N,6°44′E) is located in the south-east of the Gulf of Guinea in western Africa, 201 km east of the African continent. It consists of two large islands, São Tomé Island and Príncipe Island, and 14 small islands, such as Caroso, Pedras, Tiniosaş and Loras, in the vicinity. The country’s total land area is 1001 km^2^ (São Tomé Island: 859 km^2^; Príncipe Island: 142 km^2^) [15]. Malaria control in STP is severely hampered by economic underdevelopment and a weak public healthcare system, with the latter particularly characterized by a lack of adequate medical resources and difficulties in improving sanitary practices [1]. The present study was carried out to examine the effects of differing numbers of MDA rounds in low-transmission areas to optimize the effectiveness of MDA, optimize the use of medical resources and lessen the burden of malaria. The results showed that malaria incidence was rapidly reduced in areas with coverage rates exceeding 84% per round in both the 3-MDA and 2-MDA groups (Fig. 4). Overall, the malaria case incidence decreased steadily by 80.47% for the 3-MDA arm of the study and by 72.27% for the 2-MDA arm at 1 year after implementation. Vila Fernanda, in the 2-MDA group, had only one reported case of malaria at 1 year after implementation.

The long-term impact of MDA is a critical consideration in malaria control strategies. In low-transmission settings, MDA has been found to accelerate the elimination process. Studies have shown that China successfully eliminated malaria through MDA, and Cambodia and Thailand have explored MDA as a key tool for eliminating asymptomatic reservoirs of transmission [27]. By reducing the prevalence of *P. falciparum* carriage in children to < 1%, it was possible to interrupt malaria transmission in these regions. The results of a trial in Zambia showed that achieving local elimination of infections brought long-term benefits [21]. However, if there was incomplete clearance of *P. falciparum* carriers within the population or high population mobility, the risk of imported malaria and secondary transmission remained high, possibly undermining the long-term benefits of elimination efforts. Nevertheless, the large number of *Plasmodium* parasites killed during MDA at least reduced the risk of resistance development. To maximize the effectiveness of MDA, it is crucial to ensure high drug administration coverage, strengthen the management of imported malaria post-MDA and implement early diagnosis and treatment of suspected cases.

Adherence, defined as patients following prescribed treatments and taking medication as directed, is closely related to MDA coverage rates [4, 28]. In the present study, the adherence rates for the 3- and 2-MDA groups was 68.38% and 80.70%, respectively, indicating that longer treatment regimens were associated with lower adherence. The age stratification results showed that adherence was highest in the age group 6–13 years for both the 3-MDA and 2-MDA groups (Additional file 10: Table S10, Additional file 11: Table S11). Poor adherence was caused by several factors, such as insufficient pre-MDA training, subpar advertising and ineffective mobilization efforts, which prevented some people from actively participating in the MDA rounds because they did not comprehend its importance [29]. Some villagers were unable to receive their medications within the allotted time due to the inconvenience of the drug-taking schedule, which was mostly during school days and working hours. Additionally, school-age children (6–13 years of age) were more highly valued by their families and they were also more willing to receive MDA. Many earlier studies have shown that adherence is crucial for the success of MDA, with better adherence leading to higher MDA coverage rates and greater effectiveness of MDA [30, 31]. The sustainability of MDA highly depends on community acceptance and adherence. In the Southern Province, Zambia, the coverage of MDA declined from 70% in the first round to 57% in the second round, indicating that adherence might decrease with repeated implementation [26]. Systematic refusal to participate, such as concerns about drug side effects or doubts about the necessity of the intervention, could threaten the sustainability of MDA, particularly in scenarios requiring multi-day drug administration. In recent years, the number of malaria cases in STP has remained at around 3000, indicating an unstable state, and the number is expected to increase during the rainy season [32]. The results of the present study showed that the parasitemia and *P. falciparum* gametocytemia rates were 2.24‰ and 1.30‰ for the 3-MDA group and 2.01‰ and 1.34‰ for the 2-MDA group, respectively (Additional file 6: Table S6), indicating the presence of asymptomatic carriers in the villages. A substantial population of symptomatic carriers gradually develops because little or no attention is given to asymptomatic individuals during passive surveillance measures, and these individuals remain infectious to mosquitoes since they are unlikely to seek treatment [8]. These asymptomatic carriers increase the risk of malaria spread in the population. Although the parasite density in the blood of asymptomatic carriers may be low, they can still carry *P. falciparum* gametocytes and contribute to malaria transmission in a manner similar to symptomatic cases [33]. In areas where malaria has been eliminated or is under control, the presence of asymptomatic carriers could facilitate the resurgence of transmission and the occurrence of outbreaks. This was particularly true for individuals with a history of residence in malaria-endemic regions abroad, as they may serve as the main source of imported malaria.

Six months after MDA, microscopic examinations detected only one positive case in the 2-MDA group, which is similar to the results of Deng and colleagues using AP + PMQ during Anjouan MDA in Comoros [25]. Malaria parasite rates among randomly selected Anjouan children decreased from an average of 13.5% before MDA to 1.2%, 0.7% and 0.5% at 6, 12 and 18 months after MDA, respectively. Possible reasons for the observed decrease in the rate of parasite include: (i) the use of PMQ during MDA helped reduce the *P. falciparum* gametocytemia; (2) the rigorous 28-day follow-up of malaria cases; (iii) ongoing monitoring and treatment of the villages by technicians; (iv) randomly selected age groups of children with high adherence to medication in MDA; and (v) the continuous implementation of malaria education programs aimed at increasing parental awareness of their children's health.

Adverse events related to MDA observed in this study were consistent with the safety profile described in the first phase [15]. There were no reports of hematuria, jaundice or other hemolytic reactions with PMQ, indicating that AP + PMQ had a good safety profile and that a single low dose (0.25 mg/kg) of PMQ was well tolerated [18], consistent with previously reported results.

A number of limitations of this study need to be highlighted in the context of the study sites being on an island. Islands, due to their geographical isolation and concentrated population distribution, may offer a unique environment that enhances the effectiveness of MDA strategies. At the same time, climatic and environmental conditions on the island may also have an impact on disease transmission and treatment outcomes [34, 35]. In this context, the effects of two and three rounds of MDA were found to be comparable. This may suggest that in islands, increasing the number of MDA rounds may not result in a significant improvement in effectiveness, possibly due to limitations such as population distribution and transport. However, this does not mean that the potential of MDA in islands should be overlooked. On the contrary, there is a need to further explore ways to optimize MDA strategies to align with the unique circumstances of islands. It is worth mentioning, however, that the situation in inland regions may significantly differ from that in island regions. Inland regions may have a wider population distribution and possess a more complex and diverse geography. These differences may lead to significant variations in the effectiveness of MDA in inland regions compared to island regions. Therefore, further trials and studies need to be conducted in inland regions to determine the best MDA strategies. Further, the attendance rates of the services, the positivity rates of slides and RDTs and the evolution of the recruitment rate compared to previous years would have provided valuable insights into the dynamics of malaria transmission. However, as this large-scale MDA was the first implementation of MDA in STP, no historical data were available for comparison of these indicators. Future large-scale MDAs conducted in STP could use this study as a baseline to explore dynamic changes in transmission patterns. In addition to these factors, socioeconomic conditions, logistical constraints and cultural differences should be carefully considered, as they may significantly influence the effectiveness of MDA. For example, low-income groups often faced difficulties participating in MDA due to challenges in taking time off work, which likely contributed to uneven coverage. Similarly, the uneven distribution of drug delivery, transportation, storage and medical resources (slides and TDRs) may have compromised the implementation effectiveness, particularly in remote areas. Moreover, limited understanding of MDA in some communities might have negatively impacted participation rates and adherence, further highlighting the need for targeted community engagement and education. By considering these factors together, a more comprehensive understanding of the challenges and opportunities of MDA in different contexts can be achieved. Finally, although this trial was conducted in an island region, the results are still informative. Through further exploration and research, a better understanding of the applicability and effectiveness of MDA in different regions can be gained, thereby providing more support and guidance for global efforts to eradicate the disease. This will not only help develop more effective public health strategies but also contribute to the development of global public health.

## Conclusions

The MDA strategy effectively reduced the prevalence of malaria infections in the regions where it was implemented. High coverage and adherence were key factors contributing to its success. It is worth noting that in some regions, conducting two rounds of MDA is likely to achieve the same desired results as conducting three round of MDA. The results of this study will inform global malaria control and the reduction of the malaria burden, especially in low-transmission regions. Therefore, the implementation of MDA needs to be flexibly adapted and optimized to the local situation to ensure its effectiveness and sustainability. In the future, advancements in science and technology coupled with strengthened global health cooperation are expected to enable STP to further consolidate and expand these achievements.

## Supplementary Information

Additional file 1.

Additional file 2.

Additional file 3.

Additional file 4.

Additional file 5.

Additional file 6.

Additional file 7.

Additional file 8.

Additional file 9.

Additional file 10.

Additional file 11.

## Data Availability

No datasets were generated or analyzed during the current study.

## Abbreviations

AMPC: Average monthly percent change
AP: Artemisinin-piperaquine
AP-MDA: Mass drug administration with artemisinin-piperaquine
GSM: Grid search model
2-MDA: Two rounds of mass drug administration
3-MDA: Three rounds of mass drug administration
MDA: Mass drug administration
MPC: Monthly percent change
NCDC: National Centre for Disease Control and Prevention
PMQ: Primaquine
RDTs: Rapid diagnostic tests
STP: São Tomé and Príncipe
