# Supplementary material for: A non-randomized, open-label study to assess the impact of rounds of mass drug administration with artemisinin-piperaquine plus primaquine on malaria in São Tomé Island
Source: Parasit Vectors. 2025 May 16;18:177. doi: 10.1186/s13071-025-06768-1 (PMC12084925; doi:10.1186/s13071-025-06768-1)
Supplement: Supplementary file 1 — Additional file 1. [file 13071_2025_6768_MOESM1_ESM.docx]

**Additional file 1: Table 1. Village geographic coordinate information**

| **Name** | **Longitude(E)** | **Latitude(N)** |
| --- | --- | --- |
| **3-MDA** |  |  |
| Fundação | 6.7196908 | 0.3322117 |
| Saton | 6.7106712 | 0.3726341 |
| Atrás Cimiterio | 6.7193142 | 0.3466937 |
| Ponte Graça | 6.7230433 | 0.3392986 |
| Oquê Del Rei | 6.7124258 | 0.3487857 |
| **2-MDA** |  |  |
| Vila Fernanda | 6.7230535 | 0.3341572 |
| Atrás Cadeia | 6.7321268 | 0.3310021 |
| Pema Pema | 6.7419595 | 0.3156092 |
| Pantufo | 6.7437659 | 0.3150349 |
| Boa Morte | 6.7177572 | 0.3403204 |

Abbreviations: MDA,mass drug administration；E,east longitude;N,northern latitude.
