# Supplementary material for: A non-randomized, open-label study to assess the impact of rounds of mass drug administration with artemisinin-piperaquine plus primaquine on malaria in São Tomé Island
Source: Parasit Vectors. 2025 May 16;18:177. doi: 10.1186/s13071-025-06768-1 (PMC12084925; doi:10.1186/s13071-025-06768-1)
Supplement: Supplementary file 2 — Additional file 2. [file 13071_2025_6768_MOESM2_ESM.docx]

**Additional file 2: Table 2. Dose of Artemisinin-piperaquine and Primaquine**

**Dose of Artemisinin-piperaquine**

| **Weight(kg)** | **Total** | **Day** | |
| --- | --- | --- | --- |
|  |  | **0** | **1** |
| 5–15 | 1 tablet | $\frac{1}{2}$tablet | $\frac{1}{2}$tablet |
| 16–30 | 2 tablets | 1 tablets | 1 tablet |
| 31–45 | 3 tablets | 1$\frac{1}{2}$tablets | 1$\frac{1}{2}$tablets |
| ＞45 | 4 tablets | 2 tablets | 2 tablets |

AP：Each tablet 62.5 mg of artemisinin + 375 mg of piperaquine

**Dose of Primaquine**

| **Age(years)** | **Weight(kg)** | **Day 0** |
| --- | --- | --- |
| 6m-1 | ≤10 | $\frac{1}{3}$tablet |
| 1-4 | 11-15 | $\frac{1}{2}$tablet |
| 5-7 | 16-20 | $\frac{2}{3}$tablet |
| 8-13 | 21-30 | 1 tablet |
|  | 31-45 | 1$\frac{1}{2}$tablets |
| ≥14 | ＞45 | 2 tablets |

PMQ：Each tablet Primaquine phosphate 7.5mg
