# Supplementary material for: A non-randomized, open-label study to assess the impact of rounds of mass drug administration with artemisinin-piperaquine plus primaquine on malaria in São Tomé Island
Source: Parasit Vectors. 2025 May 16;18:177. doi: 10.1186/s13071-025-06768-1 (PMC12084925; doi:10.1186/s13071-025-06768-1)
Supplement: Supplementary file 3 — Additional file 3. [file 13071_2025_6768_MOESM3_ESM.pdf]

REPÚBLICA DEMOCRÁTICA DE SÃO TOMÉ E PRÍNCIPE 圣多美和普林西比民主共和国
MINISTÉRIO DA SAÚDE 卫生部
CENTRO NACIONAL DE FARMACOVIGILÂNCIA
国家药物不良反应中心登记表

中 国 援 助
ASSISTÊNCIA DA CHINA

FORMULÁRIO DE NOTIFICAÇÃO DE EFEITOS ADVERSOS DE MEDICAMENTOS, VACINAS E PRODUTOS PARA A SAÚDE
药物治疗及疫苗接种不良反应登记表

PACIENTE 患者信息

Nome（姓名）	Idade（年龄）	Sexo（性别）	Peso (kg)	Altura（cm）

Paciente do sexo feminine está gravida?女性患者是否怀孕：☐ Sim 是 ☐ Não 否

Data da última menstruação 末次月经时间:

☐ Tabaco(抽烟) ☐ Álcool(饮酒) ☐ Remédio tradicional (日常生活习惯)

☐ Tratamento crónico (especificar) 是否服用治疗慢性疾病药物

História médica relevante, reação ao medicamento, exames laboratoriais, comentários (adicionar uma folha adicional, se necessário) 详细描述相关病史，服药后反应，检验报告及实验室检查：

....

....

Adress 地址：..Tel 联系电话：..Ville/village 村庄：..

MEDICAMENTO(S) E/OU PRODUTO(S) TOMADO NO PERÍODO 药品相关信息

Nome comercial 药品商品名	Data de validade 有效期	Local de compra 购买地点	Modo de administração 用药方式	Dosagem 用药剂量	Período 服药周期		Motivo de administração 用药原因
					Início 开始	Fim 结束	

Item de verificação 检查项目: ☐ Decidir por si 自行用药 ☐ Recomendação por farmacêutico 药店药剂师推荐

☐ Erro terapêutico 治疗错误

Se vacina: qual?疫苗种类：..nº de lote 产品批号：vacina 疫苗批号..
solvente 溶剂批号..
Local de vacinação 疫苗接种地点：..

☐ Público 公立医院 ☐ Privado 私立医院 ☐ Campanha de vacinação 大型疫苗接种活动

REPÚBLICA DEMOCRÁTICA DE SÃO TOMÉ E PRÍNCIPE 圣多美和普林西比民主共和国
MINISTÉRIO DA SAÚDE 卫生部
CENTRO NACIONAL DE FARMACOVIGILÂNCIA
国家药物不良反应中心登记表

中 国 援 助
ASSISTÊNCIA DA CHINA

EFEITO(S) ADVERSO(S) 药物不良反应

Descrição do efeito indesejável 不良反应详细描述：

……
……
……

Data de aparecimento 出现时间： ______/______/______/

minutos 分/ horas 小时/dias 天/mês 月（marque a nota útil 请根据不良反应出现时间的长久作出适当选择）

Avaliação 现状评估

☐ Recuperado 恢复 ☐ Complicado 并发症
☐ Sequências 后遗症 ☐ Morte 死亡
☐ Ainda não curado 尚未恢复
☐ Prognóstico vital 预后不佳

Meio de tratamento 治疗手段

☐ Internamento 住院治疗
☐ Prolongamento de internamento 延长住院时间
☐ Tratamento corretivo 纠正治疗手段

Parada de medicamento 是否停药：☐ Sim 是 ☐ Não 否 Diminuição de dose de medicamento 剂量减量：Sim 是/ Não 否

Efeito adverso após de mudança de dose r 更改剂量后不良反应变化：

☐ Desaparecido 症状消失 ☐ Diminuído 症状减轻 ☐ Não aplicável 无明显变化

Se existir os efeitos adversos quando tomar novamente os medicamentos? 停药后重新用药是否再次出现不良反应？

☐ Sim 是 ☐ Não 否

Informações de efeitos adversos 不良反应上报者信息

Nome 姓名 ………………………… Telefone/Fax 联系电话/传真 ………………………………… DATA 日期 …………………………… Assinatura 签名：

Profissão 职业： ☐ Médico 医生 ☐ Farmacêutico 药剂师 ☐ Assistente médico 助理医师

☐ Parteira 助产士 ☐ Enfermeira 护士 ☐ Outros 其他

Região 地区 ……………………………… Distrito 县 ……………………………… Posto sanitário 卫生机构 ………………………………………… Sector 科室名称 ………………………………………

Informaões do paciente permanecem estritamente confidenciais

对患者信息及不良反应上报者信息进行保密协议
