# Supplementary material for: A non-randomized, open-label study to assess the impact of rounds of mass drug administration with artemisinin-piperaquine plus primaquine on malaria in São Tomé Island
Source: Parasit Vectors. 2025 May 16;18:177. doi: 10.1186/s13071-025-06768-1 (PMC12084925; doi:10.1186/s13071-025-06768-1)
Supplement: Supplementary file 4 — Additional file 4. [file 13071_2025_6768_MOESM4_ESM.docx]

**Additional file 4: Table 4 Baseline characteristics**

| **Characteristics** | **3-MDA** | **2-MDA** | **Total** | ***P*-value** |
| --- | --- | --- | --- | --- |
| Males (%) | 3924(46.34) | 4044(45.08) | 7968(45.69) | 0.097 |
| Age group (%) |  |  |  |  |
| ≤6 months | 117(1.38) | 104(1.16) | 221(1.27) | 0.199 |
| 7-12 momths | 108(1.28) | 109(1.22) | 217(1.24) | 0.733 |
| 1-5 years | 1207(14.25) | 1342(14.96) | 2549(14.62) | 0.191 |
| 6-13 years | 1901(22.45) | 2143(23.89) | 4044(23.19) | 0.025 |
| ≥14 years | 5135(60.64) | 5272(58.77) | 10407(59.68) | 0.012 |
| Registered Population | 8468 | 8970 | 17438 |  |
| Pregnant women | 231 | 211 | 442 | 0.123 |
| Severe diseases | 27 | 33 | 60 | 0.607 |
| Malaria test,  positive（/1000） |  |  |  |  |
| RDT | 61(7.20) | 41(4.57) | 102(5.85) | 0.028 |
| Microscopy | 19(2.24) | 18(2.01) | 37(2.12) | 0.745 |
| Gametocyte | 11(1.30) | 12(1.34) | 23(1.32) | ＞0.999 |

Abbreviations: MDA,mass drug administration;RDT, Malaria rapid diagnostic tests.
