# Supplementary material for: A non-randomized, open-label study to assess the impact of rounds of mass drug administration with artemisinin-piperaquine plus primaquine on malaria in São Tomé Island
Source: Parasit Vectors. 2025 May 16;18:177. doi: 10.1186/s13071-025-06768-1 (PMC12084925; doi:10.1186/s13071-025-06768-1)
Supplement: Supplementary file 5 — Additional file 5. [file 13071_2025_6768_MOESM5_ESM.docx]

**Additional file 5: Table 5. Summary of adverse events for MDA**

| **Adverse Events Numbers (%^a^)** | **3-MDA** | | | |  | **2-MDA** | | | **Total** | ***P*-value^b^** |
| --- | --- | --- | --- | --- | --- | --- | --- | --- | --- | --- |
|  | **Round 1** | **Round 2** | **Round 3** | **Total** |  | **Round 1** | **Round 2** | **Total** |  |  |
| Headache | 0.43  (29/6704) | 0.10  (7/6749) | 0.03  (2/7095) | **0.18**  **(38/20548)** |  | 0.32  (24/7430) | 0.09  (7/7935) | **0.2**  **(31/15365)** | **0.19**  **(69/35913)** | 0.573 |
| Dizziness | 0.39  (26/6704) | 0.04  (3/6749) | 0.03  (2/7095) | **0.15**  **(31/20548)** |  | 0.23  (17/7430) | 0.06  (5/7935) | **0.14**  **(22/15365)** | **0.15**  **(53/35913)** | ＞0.999 |
| Fever | 0.09  (6/6704) | 0.03  (2/6749) | 0.01  (1/7095) | **0.04**  **(9/20548)** |  | 0.05  (4/7430) | 0.05  (4/7935) | **0.05**  **(8/15365)** | **0.05**  **(17/35913)** | 0.800 |
| Nausea | 0.07  (5/6704) | 0  (0/6749) | 0  (0/7095) | **0.02**  **(5/20548)** |  | 0.04  (3/7430) | 0  (0/7935) | **0.02**  **(3/15365)** | **0.02**  **(8/35913)** | ＞0.999 |
| Vomiting | 0.21  (14/6704) | 0.03  (2/6749) | 0  (0/7095) | **0.08**  **(16/20548)** |  | 0.07  (5/7430) | 0.03  (2/7935) | **0.05**  **(7/15365)** | **0.06**  **(23/35913)** | 0.276 |
| Abdominal pain | 0.19  (13/6704) | 0.07  (5/6749) | 0  (0/7095) | **0.09**  **(18/20548)** |  | 0.16  (12/7430) | 0.03  (2/7935) | **0.09**  **(14/15365)** | **0.09**  **(32/35913)** | 0.850 |
| Weakness | 0.33  (22/6704) | 0.07  (5/6749) | 0.04  (3/7095) | **0.15**  **(30/20548)** |  | 0.23  (17/7430) | 0.04  (3/7935) | **0.13**  **(20/15365)** | **0.14**  **(50/35913)** | 0.874 |
| Others^c^ | 0.07  (5/6704) | 0.03  (2/6749) | 0  (0/7095) | **0.03**  **(7/20548)** |  | 0.07  (5/7430) | 0.01  (1/7935) | **0.04**  **(6/15365)** | **0.04**  **(13/35913)** | 0.779 |
| Total | 1.79  (120/6704) | 0.39  (26/6749) | 0.11  (8/7095) | **0.75**  **(154/20548)** |  | 1.17  (87/7430) | 0.30  (24/7935) | **0.72**  **(111/15365)** | **0.74**  **(265/35913)** | - |

Abbreviations: MDA,mass drug administration.

a:The percentage = the number of adverse events/the total population of three or two rounds of MDA. The population recorded for the 3-MDA were 6,704+6,749+7,095=20,548 person-times; the 2-MDA were 7,430+7,935=15,365 person-times; and the total number of MDA were 20,548 + 15,365 = 35,913 person-times participated in MDA.

b:P-value：Total number of adverse events in 3-MDA vs total number of adverse events in 2-MDA.

c:Others：diarrhoea, itching, muscle pain, etc.
