# Supplementary material for: A non-randomized, open-label study to assess the impact of rounds of mass drug administration with artemisinin-piperaquine plus primaquine on malaria in São Tomé Island
Source: Parasit Vectors. 2025 May 16;18:177. doi: 10.1186/s13071-025-06768-1 (PMC12084925; doi:10.1186/s13071-025-06768-1)
Supplement: Supplementary file 6 — Additional file 6. [file 13071_2025_6768_MOESM6_ESM.docx]

**Additional file 6: Table 6. Parasitemia Rates Before and post-MDA**

| **Rounds and District, Rate(/1000)** | **Pre-MDA Mar 2022** | | | | **Post-MDA Dec 2022** | | | |
| --- | --- | --- | --- | --- | --- | --- | --- | --- |
|  | **Population** | **RDT** | **Microscopy** | **Gametocyte** | **Population** | **RDT** | **Microscopy** | **Gametocyte** |
| **3-MDA** |  |  |  |  |  |  |  |  |
| Fundação | 944 | 2.12(2/944) | 4.24(4/944) | 3.18(3/944) | 100 | 0(0/100) | 0(0/100) | 0(0/100) |
| Saton | 1050 | 3.81(4/1050) | 6.67(7/1050) | 0(0/1050) | 100 | 0(0/100) | 0(0/100) | 0(0/100) |
| Atrás Cimiterio | 1127 | 17.75(20/1127) | 5.32(6/1127) | 0(0/1127) | 100 | 0(0/100) | 0(0/100) | 0(0/100) |
| Ponte Graça | 2068 | 6.77(14/2068) | 0.48(1/2068) | 1.93(4/2068) | 100 | 0(0/100) | 0(0/100) | 0(0/100) |
| Oquê Del Rei | 3279 | 6.40(21/3279) | 0.30(1/3279) | 1.22(4/3279) | 100 | 0(0/100) | 0(0/100) | 0(0/100) |
| **Total** | **8468** | **7.20(61/8468)** | **2.24(19/8468)** | **1.30(11/8468)** | **500** | **0(0/500)** | **0(0/500)** | **0(0/500)** |
| **2-MDA** |  |  |  |  |  |  |  |  |
| Vila Fernanda | 787 | 6.35(5/787) | 5.08(4/787) | 6.35(5/787) | 100 | 0(0/100) | 0(0/100) | 0(0/100) |
| Atrás Cadeia | 1290 | 3.10(4/1290) | 3.10(4/1290) | 0(0/1290) | 100 | 0(0/100) | 0(0/100) | 0(0/100) |
| Pema Pema | 1301 | 3.84(5/1301) | 3.84(5/1301) | 0.77(1/1301) | 100 | 0(0/100) | 0(0/100) | 0(0/100) |
| Pantufo | 2630 | 5.70(15/2630) | 1.14(3/2630) | 1.14(3/2630) | 100 | 0(0/100) | 10.00(1/100) | 0(0/100) |
| Boa Morte | 2962 | 4.05(12/2962) | 0.68(2/2962) | 1.01(3/2962) | 100 | 0(0/100) | 0(0/100) | 0(0/100) |
| **Total** | **8970** | **4.57(41/8970)** | **2.01(18/8970)** | **1.34(12/8970)** | **500** | **0(0/500)** | **2.00(1/500)** | **0(0/500)** |

Abbreviations: MDA,mass drug administration;RDT,Malaria rapid diagnostic tests.
