# Supplementary material for: A non-randomized, open-label study to assess the impact of rounds of mass drug administration with artemisinin-piperaquine plus primaquine on malaria in São Tomé Island
Source: Parasit Vectors. 2025 May 16;18:177. doi: 10.1186/s13071-025-06768-1 (PMC12084925; doi:10.1186/s13071-025-06768-1)
Supplement: Supplementary file 7 — Additional file 7. [file 13071_2025_6768_MOESM7_ESM.docx]

**Additional file 7: Table 7. Incidence of malaria before and post-MDA/1000**

| **Rounds and District** | **Population^a^** | **Malaria Case Incidence /1000** | | | |
| --- | --- | --- | --- | --- | --- |
|  |  | **3 years Pre-MDA Apr 2019-Mar 2020** | **2 years Pre-MDA Apr 2020-Mar 2021** | **1 year Pre-MDA Apr 2021-Mar 2022** | **1 year Post-MDA^b^** |
| **3-MDA** |  |  |  |  |  |
| Fundação | 944 | 22.17(20/902) | 42.57(39/916) | 59.14(55/930) | 11.65(11/944) |
| Saton | 1050 | 35.88(36/1003) | 25.53(26/1018) | 49.32(51/1034) | 5.71(6/1050) |
| Atrás Cimiterio | 1127 | 12.07(13/1077) | 8.23(9/1093) | 17.12(19/1110) | 9.76(11/1127) |
| Ponte Graça | 2068 | 11.64(23/1976) | 26.91(54/2006) | 35.84(73/2037) | 1.93(4/2068) |
| Oquê Del Rei | 3279 | 46.91(147/3134) | 14.77(47/3182) | 44.89(145/3230) | 10.67(35/3279) |
| **Total** | **8468** | **29.53(239/8093)** | **21.3(175/8216)** | **41.12(343/8341)** | **7.91(67/8468)** |
| **2-MDA** |  |  |  |  |  |
| Vila Fernanda | 787 | 17.29(13/752) | 30.13(23/763) | 54.19(42/775) | 1.27(1/787) |
| Atrás Cadeia | 1290 | 4.87(6/1233) | 3.99(5/1252) | 18.88(24/1271) | 9.3(12/1290) |
| Pema Pema | 1301 | 12.87(16/1243) | 1.59(2/1262) | 21.86(28/1281) | 7.69(10/1301) |
| Pantufo | 2630 | 22.67(57/2514) | 4.31(11/2552) | 13.51(35/2591) | 7.22(19/2630) |
| Boa Morte | 2962 | 8.83(25/2831) | 19.83(57/2874) | 43.52(127/2918) | 9.79(29/2962) |
| **Total** | **8970** | **13.65(117/8573)** | **11.26(98/8703)** | **28.97(256/8836)** | **7.92(71/8970)** |

Abbreviations: MDA,mass drug administration

a:Numbers of inhabitants are from 2022 the MDA registered population data,and the population is growing at a rate of approximately 1.5% per year.

b:1 year Post-MDA:3-MDA is from Jul 2022 to Jun 2023;2-MDA is from Jun 2022 to May 2023.
