# Supplementary material for: A non-randomized, open-label study to assess the impact of rounds of mass drug administration with artemisinin-piperaquine plus primaquine on malaria in São Tomé Island
Source: Parasit Vectors. 2025 May 16;18:177. doi: 10.1186/s13071-025-06768-1 (PMC12084925; doi:10.1186/s13071-025-06768-1)
Supplement: Supplementary file 8 — Additional file 8. [file 13071_2025_6768_MOESM8_ESM.docx]

**Additional file 8: Table 8. Trends in malaria incidence in Joinpoint(Jan. 2019-Jun. 2023)**

| **Rounds and District** | **Time period** | **MPC (95%CI)** | ***t value*** | ***P value*** | **AMPC (95%CI)** | ***t value*** | ***P value*** |
| --- | --- | --- | --- | --- | --- | --- | --- |
| **3-MDA** | Jan. 2019-Aug. 2019 | -30.39  (-46.93~-8.71) | -2.69 | 0.010^*^ | -7.09  (-11.23~-2.77) | -3.17 | 0.002^**^ |
|  | Aug. 2019-Mar. 2022 | 3.35  (0.27~6.53) | 2.19 | 0.033^*^ |  |  |  |
|  | Mar. 2022-Jun. 2023 | -14.71  (-21.72~-7.07) | -3.73 | 0.001^**^ |  |  |  |
| **2-MDA** | Jan. 2019-Feb. 2022 | 1.93  (-0.50~4.22) | 1.59 | 0.117 | -1.65  (-4.55~1.34) | -1.09 | 0.277 |
|  | Feb. 2022-Jun. 2023 | -9.46  (-16.84~-1.42) | -2.35 | 0.023^*^ |  |  |  |

Abbreviations: MDA,mass drug administration

**P*<0.05 ***P*<0.01
