# Supplementary material for: A non-randomized, open-label study to assess the impact of rounds of mass drug administration with artemisinin-piperaquine plus primaquine on malaria in São Tomé Island
Source: Parasit Vectors. 2025 May 16;18:177. doi: 10.1186/s13071-025-06768-1 (PMC12084925; doi:10.1186/s13071-025-06768-1)
Supplement: Supplementary file 9 — Additional file 9. [file 13071_2025_6768_MOESM9_ESM.docx]

**Additional file 9: Table 9. Incidence of malaria post-MDA/1000**

| **Rounds and District** | **Malaria Case Incidence /1000** | | | | |
| --- | --- | --- | --- | --- | --- |
|  | **3 mo Pre-MDA Jan -Mar 2022** | **3 mo Post-MDA^a^** | **6 mo Post-MDA^b^** | **9 mo Post-MDA^c^** | **12 mo Post-MDA^d^** |
| **3-MDA** |  |  |  |  |  |
| Fundação | 12.71 (12/944) | 2.12 (2/944) | 2.12 (2/944) | 4.18 (4/958) | 3.13 (3/958) |
| Saton | 9.52 (10/1050) | 2.86 (3/1050) | 0.95 (1/1050) | 0 (0/1066) | 1.88 (2/1066) |
| Atrás Cimiterio | 1.77 (2/1127) | 3.55 (4/1127) | 1.77 (2/1127) | 1.75 (2/1144) | 2.62 (3/1144) |
| Ponte Graça | 13.54 (28/2068) | 0.48 (1/2068) | 0 (0/2068) | 0 (0/2099) | 1.43 (3/2099) |
| Oquê Del Rei | 15.86 (52/3279) | 5.79 (19/3279) | 1.52 (5/3279) | 2.1 (7/3328) | 1.2 (4/3328) |
| **Total** | **12.28 (104/8468)** | **3.42 (29/8468)** | **1.18 (10/8468)** | **1.51 (13/8595)** | **1.75 (15/8595)** |
| **2-MDA** |  |  |  |  |  |
| Vila Fernanda | 11.44 (9/787) | 0 (0/787) | 1.27 (1/787) | 0 (0/799) | 0 (0/799) |
| Atrás Cadeia | 8.53 (11/1290) | 8.53 (11/1290) | 0.78 (1/1290) | 0 (0/1309) | 0 (0/1309) |
| Pema Pema | 6.15 (8/1301) | 0.77 (1/1301) | 2.31 (3/1301) | 1.51 (2/1321) | 3.03 (4/1321) |
| Pantufo | 6.46 (17/2630) | 4.56 (12/2630) | 0 (0/2630) | 0.37 (1/2669) | 2.25 (6/2669) |
| Boa Morte | 5.74 (17/2962) | 3.04 (9/2962) | 2.03 (6/2962) | 2.00 (6/3006) | 2.66 (8/3006) |
| **Total** | **6.91 (62/8970)** | **3.68 (33/8970)** | **1.23 (11/8970)** | **0.99 (9/9104)** | **1.98 (18/9104)** |

Abbreviations: MDA,mass drug administration

a:3 mo Post-MDA:3-MDA is from Jul. to Sept. 2022;2-MDA is from Jun. to Aug. 2022.

b:6 mo Post-MDA:3-MDA is from Oct. to Dec. 2022;2-MDA is from Sept. 2022 to Nov. 2022.

c:9 mo Post-MDA:3-MDA is from Jan. to Mar. 2023;2-MDA is from Dec. 2022 to Feb. 2023.

d:12 mo Post-MDA:3-MDA is from Apr. to Jun. 2023;2-MDA is from Mar. 2023 to May 2023.
