# Supplementary material for: A non-randomized, open-label study to assess the impact of rounds of mass drug administration with artemisinin-piperaquine plus primaquine on malaria in São Tomé Island
Source: Parasit Vectors. 2025 May 16;18:177. doi: 10.1186/s13071-025-06768-1 (PMC12084925; doi:10.1186/s13071-025-06768-1)
Supplement: Supplementary file 10 — Additional file 10. [file 13071_2025_6768_MOESM10_ESM.docx]

**Additional file 10: Table 10. Complete course of treatment rate(Age Groups)**

| **Rounds and District** | **Complete course of treatment rate (%)^*^** | | | |
| --- | --- | --- | --- | --- |
|  | **7-12 months** | **1-5 years** | **6-13 years** | **≥14 years** |
| **3-MDA** |  |  |  |  |
| Fundação | 0.67(6/891) | 12.23(109/891) | 17.85(159/891) | 39.06(348/891) |
| Saton | 1.48(15/1011) | 10.19(103/1011) | 19.19(194/1011) | 44.31(448/1011) |
| Atrás Cimiterio | 0.57(6/1051) | 11.13(117/1051) | 16.56(174/1051) | 39.49(415/1051) |
| Ponte Graça | 0.71(14/1966) | 9.16(180/1966) | 16.79(330/1966) | 38.40(755/1966) |
| Oquê Del Rei | 1.05(32/3040) | 10.82(329/3040) | 15.95(485/3040) | 40.23(1223/3040) |
| **Total** | **0.92(73/7959)** | **10.53(838/7959)** | **16.86(1342/7959)** | **40.07(3189/7959)** |
| **2-MDA** |  |  |  |  |
| Vila Fernanda | 0.54(4/745) | 11.14(83/745) | 17.72(132/745) | 54.23(404/745) |
| Atrás Cadeia | 0.57(7/1234) | 9.81(121/1234) | 17.75(219/1234) | 51.30(633/1234) |
| Pema Pema | 1.39(17/1225) | 12.33(151/1225) | 21.39(262/1225) | 42.94(526/1225) |
| Pantufo | 1.21(30/2487) | 16.28(405/2487) | 23.56(586/2487) | 45.20(1124/2487) |
| Boa Morte | 0.85(24/2820) | 12.34(348/2820) | 20.60(581/2820) | 42.94(1211/2820) |
| **Total** | **0.96(82/8511)** | **13.02(1108/8511)** | **20.91(1780/8511)** | **45.80(3898/8511)** |

Abbreviations: MDA, mass drug administration.

*Complete course of treatment rate(Age Groups)=(No. complete course of treatment participated in each age group)/(Inclusion of total population)*100%
