# Supplementary material for: A non-randomized, open-label study to assess the impact of rounds of mass drug administration with artemisinin-piperaquine plus primaquine on malaria in São Tomé Island
Source: Parasit Vectors. 2025 May 16;18:177. doi: 10.1186/s13071-025-06768-1 (PMC12084925; doi:10.1186/s13071-025-06768-1)
Supplement: Supplementary file 11 — Additional file 11. [file 13071_2025_6768_MOESM11_ESM.docx]

**Additional file 11: Table 11. Compliance rates by age group**

| **Rounds and District** | **Compliance rates(%)** | | | |
| --- | --- | --- | --- | --- |
|  | **7-12 momths** | **1-5 years** | **6-13 years** | **≥14 years** |
| **3-MDA** |  |  |  |  |
| Fundação | 60.00(6/10) | 78.42(109/139) | 75.36(159/211) | 65.78(348/529) |
| Saton | 88.24(15/17) | 82.40(103/125) | 80.83(194/240) | 71.45(448/627) |
| Atrás Cimiterio | 54.55(6/11) | 65.73(117/178) | 69.88(174/249) | 68.48(415/606) |
| Ponte Graça | 56.00(14/25) | 66.18(180/272) | 67.07(330/492) | 64.20(755/1176) |
| Oquê Del Rei | 80.00(32/40) | 68.12(329/483) | 70.49(485/688) | 66.90(1223/1828) |
| **Total** | **70.87(73/103)** | **70.01(838/1197)** | **71.38(1342/1880)** | **66.91(3189/4766)** |
| **2-MDA** |  |  |  |  |
| Vila Fernanda | 66.67(4/6) | 86.46(83/96) | 81.99(132/161) | 84.34(404/479) |
| Atrás Cadeia | 87.50(7/8) | 81.76(121/148) | 79.06(219/277) | 79.22(633/799) |
| Pema Pema | 73.91(17/23) | 81.62(151/185) | 81.11(262/323) | 76.12(526/691) |
| Pantufo | 85.71(30/35) | 87.10(405/465) | 89.60(586/654) | 84.38(1124/1332) |
| Boa Morte | 68.57(24/35) | 78.91(348/441) | 80.47(581/722) | 74.89(1211/1617) |
| **Total** | **76.64(82/107)** | **83.00(1108/1335)** | **83.29(1780/2137)** | **79.26(3898/4918)** |

Abbreviations: MDA,mass drug administration.

*Compliance rates by age group=(No. complete course of treatment participated in each age group)/(Inclusion of total population in each group)*100%
